# Callous-unemotional traits in youth with autism spectrum disorder (ASD): replication of prevalence estimates and associations with gaze patterns when viewing fearful faces

**DOI:** 10.1017/S0954579420000449

**Published:** 2021-10

**Authors:** Virginia Carter Leno, Rachael Bedford, Susie Chandler, Pippa White, Isabel Yorke, Tony Charman, Andrew Pickles, Emily Simonoff

**Affiliations:** 1 Department of Biostatistics and Health Informatics, Institute of Psychiatry, Psychology & Neuroscience, King's College London, London, UK; 2 Department of Child and Adolescent Psychiatry, Institute of Psychiatry, Psychology & Neuroscience, King's College London, London, UK; 3 Department of Psychology, Institute of Psychiatry, Psychology & Neuroscience, King's College London, London, UK; 4 South London and Maudsley NHS Foundation Trust (SLaM), London, UK; 5 Maudsley Biomedical Research Centre for Mental Health, London, UK

**Keywords:** autism, callous-unemotional traits, conduct problems, fear recognition, QUEST

## Abstract

Research suggests an increased prevalence of callous-unemotional (CU) traits in children with autism spectrum disorder (ASD), and a similar impairment in fear recognition to that reported in non-ASD populations. However, past work has used measures not specifically designed to measure CU traits and has not examined whether decreased attention to the eyes reported in non-ASD populations is also present in individuals with ASD. The current paper uses a measure specifically designed to measure CU traits to estimate prevalence in a large community-based ASD sample. Parents of 189 adolescents with ASD completed questionnaires assessing CU traits, and emotional and behavioral problems. A subset of participants completed a novel emotion recognition task (*n* = 46). Accuracy, reaction time, total looking time, and number of fixations to the eyes and mouth were measured. Twenty-two percent of youth with ASD scored above a cut-off expected to identify the top 6% of CU scores. CU traits were associated with longer reaction times to identify fear and fewer fixations to the eyes relative to the mouth during the viewing of fearful faces. No associations were found with accuracy or total looking time. Results suggest the mechanisms that underpin CU traits may be similar between ASD and non-ASD populations.

## Introduction

Autism spectrum disorder (ASD) is a lifelong neurodevelopmental condition characterized by impairments in social communication abilities and the presence of restricted and repetitive behaviors and interests and sensory differences (American Psychiatric Association, 2013) which occurs in around 1 in 59 children (based on most recent estimates from the Centers for Disease Control and Prevention (CDC); Baio et al., 2018). Youth with ASD exhibit increased rates of behavioral problems (Kaat & Lecavalier, 2013) and callous-unemotional (CU) traits (Carter Leno et al., 2015; Rogers, Viding, James Blair, Frith, & Happé, 2006). CU traits are characterized by a lack of guilt and empathy and a tendency to use others for one's own gain (Frick & White, 2008). In non-ASD populations, this group of traits is often found in individuals who exhibit persistent and severe patterns of antisocial and aggressive behavior, and similarly within ASD populations demarcates individuals who have a more severe behavioral phenotype, with higher levels of peer problems and externalizing behaviors (although the association with conduct problems may be weaker than is reported in non-ASD populations) (Carter Leno et al., 2015). Given the reported negative outcomes for non-ASD youth with CU traits (e.g., delinquency, antisocial behavior; McMahon, Witkiewitz, & Kotler, 2010), better understanding of the manifestation and mechanisms of CU traits in ASD populations is required to promote positive outcomes.

CU traits have a prevalence rate of around 5% in typically developing general population samples (Collishaw, Maughan, Goodman, & Pickles, 2004; Fontaine, McCrory, Boivin, Moffitt, & Viding, 2011), but are found to have an elevated prevalence rate in ASD populations, with estimates of 36–51% in adolescent ASD samples (Carter Leno et al., 2015; Rogers et al., 2006). However, these studies used the Antisocial Process Screening Device (APSD; Frick & Hare, 2001) CU trait subscale, which only consists of six items and has been shown to have low internal reliability (Dadds, Fraser, Frost, & Hawes, 2005). Newer measures specifically designed to measure CU traits are now available (e.g., the Inventory of Callous Unemotional Traits; Frick, 2004) but have not yet been used to estimate the prevalence of CU traits in ASD populations.

In terms of cognitive profiles, CU traits in non-ASD populations are associated with a specific impairment in recognizing negative emotions, with the strongest effects for fear (Marsh & Blair, 2008; Muñoz, 2009; White et al., 2016) (although see Dawel, O'Kearney, McKone, & Palermo, 2012). It has been postulated that this inability to recognize affective signals of distress underlies atypical empathic development and social behavior (Blair, 2005). This is in contrast to ASD, where global impairments in the cognitive elements of interpersonal understanding (e.g., theory of mind) are thought to underpin difficulties in social communication (Jones et al., 2018; Lerner, Hutchins, & Prelock, 2011; Shimoni, Weizman, Yoran, & Raviv, 2012). Comparative studies find that the two conditions can be differentiated on the basis of the nature of their specific socio-cognitive impairments (e.g., impairments in theory of mind are found in individuals with ASD but not individuals with CU traits, whereas impairments in affective empathy are found in CU but not ASD) (Jones, Happé, Gilbert, Burnett, & Viding, 2010; Lockwood, Bird, Bridge, & Viding, 2013; Schwenck et al., 2012).

With regards to the presentation of CU traits *within* ASD populations, previous research suggests that CU traits may have a similar cognitive profile as is found in non-ASD populations, as CU traits are associated with specific impairments in recognizing negative facial expressions (fear, sadness) in individuals with ASD (Carter Leno et al., 2015; Rogers et al., 2006). In non-ASD populations, it has been proposed that decreased attention to the eye region underlies the fear recognition impairment found in individuals with high CU traits, as CU traits in adolescents are associated with poorer fear recognition and reduced looking to the eyes (Dadds, El Masry, Wimalaweera, & Guastella, 2008) and cueing to the eyes improves performance (Dadds et al., 2006). These atypicalities in face scanning may be present from an early age, as one study found infants who looked less towards their mother's faces in early infancy had higher levels of CU traits in toddlerhood (Bedford, Pickles, Sharp, Wright, & Hill, 2015). However, to our knowledge, no study has tested whether similar attentional biases are present *within* ASD populations. Infants at familial risk for ASD and individuals who meet diagnostic criteria are also found to spend less time looking at the eyes when viewing faces (Jones & Klin, 2013; Papagiannopoulou, Chitty, Hermens, Hickie, & Lagopoulos, 2014) and spend less time looking to the face when interacting with others in real-life settings (Merin, Young, Ozonoff, & Rogers, 2007; Noris, Nadel, Barker, Hadjikhani, & Billard, 2012), although recent work with larger samples report no differences in time spent looking at the eyes between toddlers with and without ASD (Kwon, Moore, Barnes, Cha, & Pierce, 2019). The manifestation of a “double hit” of ASD and CU traits is unknown; it may be the case that CU traits are not associated with atypical gaze if individuals with ASD are already spending less time looking towards the eye area. Examining the attentional biases that may underlie impoverished fear recognition, and whether they are similar in ASD versus non-ASD populations, is a key step in determining the comparability of CU traits in individuals with and without ASD. Understanding this comparability is important with regards to appraising the likely suitability of interventions developed in non-ASD populations for individuals with ASD.

Previous work in both CU and ASD populations has primarily relied on static faces to assess emotion recognition, which have been questioned for their ecological validity. Dynamic expressions are thought to contain more information than a static face, and elicit improvements in recognition accuracy in adults (Krumhuber, Kappas, & Manstead, 2013).

However, this effect appears to be diluted or not present in ASD populations (Back, Ropar, & Mitchell, 2007; Gepner, Deruelle, & Grynfeltt, 2001; Tardif, Lainé, Rodriguez, & Gepner, 2007). Comparison of stimuli presentation in non-ASD populations finds adolescents with CU traits spend less time looking at the eyes, but only for surprised expressions, and this effect was present regardless of stimulus presentation (static vs. dynamic) (Martin-Key, Graf, Adams, & Fairchild, 2018).

### Aims

This paper estimates the prevalence of CU traits, using a newer and more comprehensive measure, in a well-characterized community sample of youth with ASD. We describe additional psychiatric symptoms and other functional outcomes associated with CU traits. Using a novel and more ecologically valid dynamic emotion recognition task, we test whether CU traits are associated with impairment in recognition and reduced looking to the eyes for fearful faces, as is found in non-ASD populations**.**

## Methods

### Participants

Participants were part of the QUEST follow-up study (Salazar et al., 2015), a longitudinal community sample recruited at age 4–8 years (Wave 1; *N* = 277) and followed up throughout childhood as part of the IAMHealth project (http://iamhealthkcl.net/). See Supplementary Material for a more detailed description and a flowchart of study recruitment and participation. Upon entry to the study, participants were split into an “intensively studied” (intensive; *n* = 101) and “extensively studied” group (extensive; *n* = 176). All participating girls were invited into the intensive subsample to allow for sex comparisons, as well as a random selection of boys, stratified to provide equal numbers on IQ (</≥70), borough (inner/outer London), age (</≥6.8 years) and Social Communication Questionnaire (SCQ) score (</≥22). Although all participants had a clinical diagnosis of ASD, the intensive group had their diagnosis confirmed at Wave 2 of data collection (aged 11–15 years) with the Autism Diagnostic Observation Schedule-2 (ADOS-2; Lord et al., 2012), and the Autism Diagnostic Interview-Revised (ADI-R; Rutter, Le Couteur, & Lord, 2003). This paper reports questionnaire data from the full sample at Wave 2 (*n* = 211), data from direct assessments from the intensive group only (*n* = 83) and cognitive data from a subsample of participants from the intensive group who completed the emotion recognition task (*n* = 46). The study was approved by Camden and King's Cross Ethics Sub-Committee (14/LO/2098); parents/caregivers gave their written informed consent and children gave their assent. Table 1 gives demographic information.

**Table 1.** Sample descriptives for full sample and split by subsample

Mean (*SD*) (Range)	Total sample (*n* = 211)	Extensive subsample (*n* = 128)	Intensive subsample (*n* = 83)	Subsample who did emotion recognition task (*n* = 46)	*p* value for extensive versus intensive contrast	*p* value for intensive versus task subsample contrast
Age	13.51 (1.12) (11.2–15.8)	13.55 (1.13) (11.2–15.8)	13.45 (1.12) (11.4–15.7)	13.42 (1.13) (11.4–15.7)	.52	.88
% Male	80%	95%	57%	57%	**<.01**	.95
IQ at Wave 1	72.50 (27.43) (19–129)	77.46 (25.16) (19–129)	65.02 (29.11) (19–120)	75.66 (22.27) (24–120)	**<.01**	**<.01**
IQ	–	–	67.82 (32.40) (19–129)	79.26 (25.34) (23–129)	–	**<.01**
ADOS severity score	–	–	6.57 (2.61) (1–10)	6.80 (2.55) (1–10)	–	.54
SCQ total	17.70 (6.92) (3–35)	17.98 (7.17) (3–35)	17.24 (6.51) (4–31)	16.67 (5.77) (5–27)	.46	**<.01**
SDQ total	17.11 (6.35) (2–34)	17.76 (6.49) (3–34)	16.01 (5.99) (2–29)	15.59 (6.14) (2–28)	.06	.08
SDQ emotional problems	4.16 (2.65) (0–10)	4.31 (2.75) (0–10)	3.92 (2.48) (0–10)	4.02 (2.52) (0–10)	.32	.79
SDQ conduct problems	2.32 (1.84) (0–8)	2.44 (1.93) (0–8)	2.12 (1.67) (0–8)	1.97 (1.52) (0–6)	.24	.54
SDQ ADHD symptoms	5.94 (2.62) (0–10)	6.28 (2.68) (0–10)	5.36 (2.42) (0–10)	5.14 (2.58) (0–10)	**.02**	.57
SDQ peer problems	4.69 (2.26) (0–10)	4.74 (2.18) (0–10)	4.61 (2.40) (0–10)	4.45 (2.47) (0–10)	.71	.68
SDQ prosocial behavior	5.58 (2.78) (0–10)	5.83 (2.69) (0–10)	5.15 (2.89) (0–10)	5.77 (2.60) (0–10)	.09	.12
ICU total	28.74 (11.56) (4–68)	28.85 (11.11) (6–68)	28.56 (12.3) (4–60)	25.74 (12.49) (4–60)	.87	.15

*Note*: Unless otherwise specified, all descriptives are taken from Wave 2 of data collection. ADOS, Autism Diagnostic Observation Schedule; SCQ, Social Communication Questionnaire; SDQ, Strengths and Difficulties Questionnaire; ICU, Inventory of Callous Unemotional Traits.

### Measures

#### Parent-rated questionnaires

##### ASD severity

The SCQ – Current Version (Rutter, Bailey, & Lord, 2003) was used to assess current ASD symptom severity. Statements are scored according to whether certain difficulties have been observed in the last three months.

##### Psychiatric symptoms

CU traits were measured using the Inventory of Callous-Unemotional Traits (ICU; Frick, 2004). The full 24-item ICU was used in the intensive subsample (potential range 0–60), whereas a shorter version, consisting of eight selected items based on both clinician judgement and criteria for the DSM CU specifier (American Psychiatric Association, 2013), was used in the extensive subsample (potential range 0–24). The correlation between the short- and long-form ICU was high (*r* = .91 in the intensive sample). Total scores on the full 24-item ICU were calculated for the full sample using multiple imputation (100 imputations), with short-form ICU, SCQ and SDQ total, along with stratification variables from Wave 1 (IQ, borough, age, sex and SCQ score) included in the model. Imputations were only calculated where all variables included in the model were present, giving a full ICU score on 189/211 participants. Unless indicated, all estimates provided for the full QUEST sample are based on the aggregated test statistic across the imputed datasets. For binary classification, participants who scored ≥39 (equal to or above a T-score of 65, comparable to the top 6%, based on estimates in Viding, Simmonds, Petrides, & Frederickson, 2009) were identified as having high levels of CU traits. Internal consistency was good in the current sample (α = 0.88), similar to reported elsewhere (Essau, Sasagawa, & Frick, 2006; Viding et al., 2009).

The parent-rated Strengths and Difficulties Questionnaire (SDQ; Goodman, Ford, Simmons, Gatward, & Meltzer, 2000) was used to measure psychiatric symptoms. The SDQ comprises three psychiatric subscales of hyperactivity/inattention (attention-deficit/hyperactivity disorder (ADHD) symptoms), conduct, and emotional problems, with additional subscales of peer-relationship problems and prosocial behavior. For binary classification, we used the general population-defined cut-off ≥4 on the conduct subscale for “definite” conduct problems.

#### Direct assessments (intensive subsample only)

##### ASD symptoms

The ADOS-2 (Lord et al., 2012) is a semi-structured assessment which is considered a gold-standard instrument for assessing current ASD symptoms. A calibrated severity score is calculated, scored 0–10, which takes into account age and language level (Shumway et al., 2012). Dependent upon verbal ability, participants were assessed with either the ADOS-2 Module 1, 2, or 3.

##### Cognitive ability

IQ was estimated using one or more of the following tests: the Wechsler Abbreviated Scale of Intelligence-2 (*n* = 50; WASI; Wechsler, 2011), the Wechsler Preschool and Primary Scale of Intelligence-4 (*n* = 11; WPPSI; Wechsler, 2012) and the Mullen Scales of Early Learning (*n* = 16; MSEL; Mullen, 1997). As the WPPSI and MSEL were used out of age range, age-equivalents were calculated, and a ratio IQ derived [ratio IQ = (age-equivalent/chronological age) × 100]. Those with an MSEL ratio IQ < 20 were assigned an IQ of 19 to reflect their very low ability. On the WPPSI and the MSEL we calculated “non-verbal ability” and a “verbal ability” which were treated as analogous to the verbal IQ and performance IQ scores from the WASI.

#### Emotion recognition paradigm

##### Apparatus

Looking behavior was recorded using a Tobii TX-300 eye-tracker. Stimuli were presented using Tobii Studio and gaze data were recorded at 120 Hz. A five-point calibration sequence was run before beginning the task.

##### Stimuli

**Figure 1.**
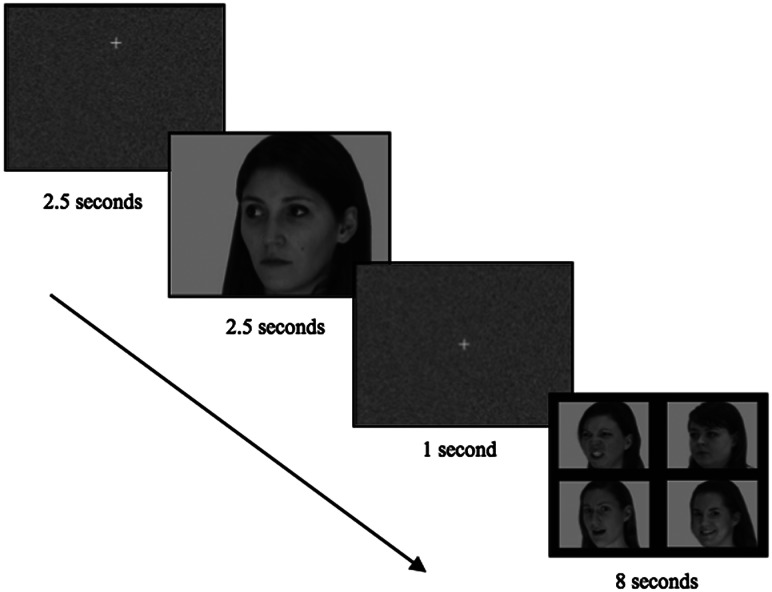
Schematic of emotion recognition paradigm.

The task was adapted from Bedford et al. (under review). Each trial consisted of an initial fixation cross (this appeared at the top of the screen for 50% of trials and at the bottom of the screen for 50% of trials to prevent incidental cuing effects) on a scrambled background (2.5 s), followed by a video of a female actor portraying a specific emotion (2.5 s; 1.5 s of motion, followed by a 1 s freeze-frame static image of the expression), a centrally presented fixation cross on a scrambled background (1 s), and then a response screen displaying four static pictures of actors portraying different emotions (8 s) (see Figure 1). See Supplementary Material for additional details. Participants were instructed to use the mouse to select the picture of the emotion that matched the emotion they had seen portrayed in the preceding video. Participants who had difficulty using the mouse were instructed to point to the matching emotion on the screen and the experimenter would click the mouse for them (*n* = 11). Five emotions (happiness, sadness, anger, fear, and neutral) were presented four times (50% with averted gaze and 50% with direct gaze), giving 20 trials in total. The order of trial presentation was randomized.

#### Behavioral data

Accuracy and reaction time (RT) were collected. An accuracy proportion for each emotion was calculated, which gave an ordinal variable coded 0, 0.25, 0.33, 0.5, 0.67, 0.75, and 1. Trials were excluded if no gaze samples were collected during initial stimulus presentation (i.e., the participant had not been paying attention to the stimulus).

Additionally, trials were excluded if no response was made during the choice screen after successful viewing of the dynamic video (as indicated by valid gaze data being collected during stimulus presentation). RT was collected for correct trials only. Participants who did not make any response throughout the task were excluded from analyses (*n* = 5).

#### Gaze data

Within each trial, two rectangular areas of interest (AOIs) were defined around the eyes and mouth using Tobii Studio. Total looking time and number of fixations were extracted for these AOIs, and for the stimuli overall. Eyes:mouth (E:M) ratio was calculated as eyes − mouth/eyes + mouth, where a higher score indicated more attention towards the eye as compared to mouth area. Trials were excluded if <70% of gaze samples were collected during stimulus presentation. An additional four participants who had <20% of usable trials were excluded from gaze analyses (*n* = 4).

### Statistical analysis

All analyses were conducted in Stata 15. Prevalence estimates were calculated using the recommended cut-off (≥39; Viding et al., 2009), and the association between binary variables of ±high levels of conduct problems and ±CU traits was tested using logistic regression. The tabulated association between binary variables was conducted in addition to continuous analyses to give a clearer illustration of the association between CU and conduct problems, and to allow comparison with previous literature. Associations between continuous CU traits and individual characteristics were tested using linear regression, and were covaried for ASD severity to identify associations above those accounted for by ASD. Here the SCQ was used, as this was available for the full sample. Associations between CU traits and performance on the emotion recognition task were tested using mixed-effects models. Accuracy was analyzed with an ordinal model with a logit link function and unstructured correlation matrix. RT, total looking time E:M and number of fixations E:M were analyzed using a gaussian model with identity link function and unstructured correlation matrix. As this paper sought to replicate the reportedly specific association between CU traits and impairments in fear recognition, performance was collapsed across all other emotions (neutral, sad, happy, angry) into one category and compared against performance for fearful faces only. The main effects of emotion (fear vs. other) and CU traits, and the interaction between the two (CU-by-emotion) were tested. As before, analyses were adjusted for ASD severity, using the ADOS calibrated severity score as it was collected from all intensive participants and provided an independent measure in addition to parent-rated measures of behavior. IQ was also included as a covariate, and for the behavioral data only, whether the experimenter had helped the participant with the mouse. Partial correlations between total looking time E:M and number of fixations E:M and accuracy and RT were calculated, accounting for experimenter help with the mouse. The margins/marginsplot commands were used to plot significant associations.

## Results

### Prevalence

Twenty-two percent (95% confidence intervals (CIs) 14%–30%) of youth with ASD scored above the designated cut-off for CU traits. In binary analyses, 44% (17/39) in the high CU group scored above the conduct problems threshold on the SDQ, in contrast to 16% (24/150) in the low CU group. The difference in rates of those scoring above the conduct problems threshold between the CU±groups was significant (*p* < .01).

### Behavioral associations

See Table 2 for bivariate associations between CU traits and individual characteristics. There were no associations between CU traits and age (*p* = .83), sex (*p* = .59), or emotional problems (*p* = .95). Higher CU traits were associated with lower full-scale, performance and verbal IQ, more severe ASD symptoms, higher levels of conduct problems, ADHD symptoms and peer problems, and lower levels of prosocial behavior (all *ps* < .05). When adjusting for ASD severity, associations with conduct problems, peer problems, and prosocial behavior remained, whereas the associations with performance IQ (*p* = .07) and ADHD symptoms (*p* = .06) became nonsignificant.

**Table 2.** Associations between callous-unemotional (CU) traits and individual characteristics

Variable	Unadjusted association with ICU total score		Adjusted for autism severity using ADOS calibrated severity score	
	β coefficient	*p* value	β coefficient	*p* value
Age	−.01	.83	.01	.73
Sex	−.01	.59	−.01	.78
Full-scale IQ^a^ (*n* = 67)	**−.95**	**<.01**	−.51	.14
Verbal IQ^a^ (*n* = 67)	**−1.01**	**<.01**	−.67	.07
Performance IQ^a^ (*n* = 67)	**−.89**	**<.01**	−.36	.30
ADOS severity score^a^ (*n* = 66)	**.06**	**.02**	—	—
SCQ total	**.31**	**<.01**	—	—
SDQ parent emotional problems	−.01	.95	−.03	.23
SDQ parent conduct problems	**.06**	**<.01**	**.04**	**<.01**
SDQ parent ADHD symptoms	**.08**	**<.01**	.04	.06
SDQ parent peer problems	**.07**	**<.01**	**.04**	**.02**
SDQ parent prosocial behavior	**−.15**	**<.01**	**−.10**	**<.01**

*Note*: ADOS, Autism Diagnostic Interview Schedule; SCQ, Social Communication Questionnaire; ICU, Inventory of Callous-Unemotional Traits; SDQ, Strengths and Difficulties Questionnaire.

a Denotes measures collected only in intensive subsample. Elsewhere full sample included in analyses (*n* = 189).

### Emotion recognition

#### Accuracy

See Table 3 for summary statistics. There was a negative main effect of emotion in which participants demonstrated lower accuracy on fearful faces than other emotions (*b* = −1.47, *p* < .01; accuracy for fear = .71, all other emotions = .86) but no effect of CU traits (*b* = −.03, *p* = .29). The CU-by-emotion interaction term was not significant (*b* = .02, *p* = .59). There was no effect of ASD severity (*b* = −.10, *p* = .53) or whether the experimenter helped with the mouse (*b* = .12, *p* = .90). IQ was at a trend level of significance (*b* = .04, *p* = .08).

**Table 3.** Average performance on emotion recognition task across total sample

Mean (*SD*) (range)	Fear	Other emotions^a^
Reaction time (RT) (seconds) (*n* = 38)	3.81 (1.33) (1.49–6.94)	2.97 (.76) (1.79–4.74)
Accuracy (average proportion correct) (*n* = 41)	.71 (.33) (0–1)	.86 (.19) (0.25–1)
Total looking time E:M (*n* = 37)	−.21 (.34) (−.84–.50)	−.17 (.35) (−.84–.46)
Number of fixations E:M (*n* = 37)	2.90 (2.31) (−.61–7.33)	2.87 (2.03) (−.68–6.61)

*Notes*: E:M indicates eyes:mouth ratio, where a higher score indicates more attention towards the eye as compared to mouth area.

a Denotes the average score collapsed across happiness, sadness, anger and neutral conditions.

#### RT

**Figure 2.**
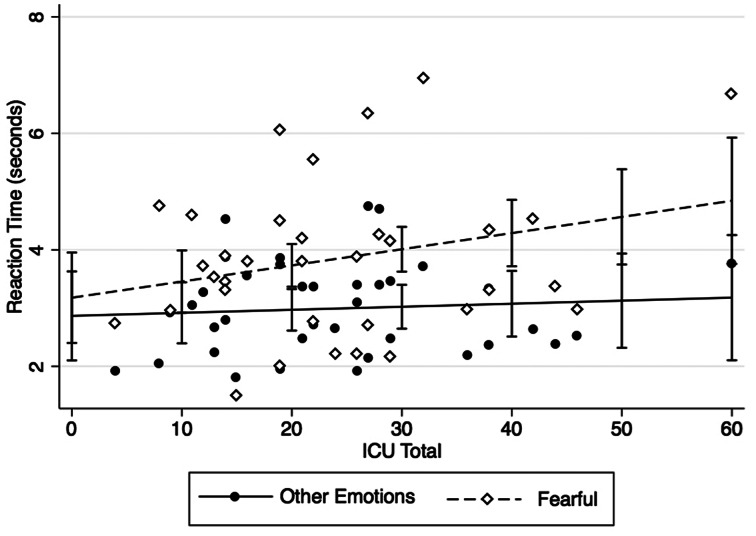
Callous-unemotional (CU) traits in adolescents with autism spectrum disorder (ASD) are associated with increased reaction time to successfully identify fear.

There was a positive main effect of emotion in which participants had longer RTs for fearful faces (*b* = .87, *p* < .01; RT for fear = 3.81 s, all other emotions = 2.97 s) but no main effect of CU traits (*b* = .02, *p* = .11). There was no effect of IQ (*b* = .01, *p* = .57) or ASD severity (*b* = .03, *p* = .54), but a significant effect of whether the experimenter helped with the mouse (*b* = 1.08, *p* < .01). The interaction term (CU-by-emotion) was significant (b = .02, *p* = .02), showing that individuals with higher CU traits had longer RTs to fearful faces only (see Figure 2).

### Total looking time E:M

There was no main effect of emotion (*b* = −.04, *p* = .40) or CU traits (*b* = −.01, *p* = .91), and the CU-by-emotion interaction term was not significant (*b* = .01, *p* = .49). The effects of IQ and ASD severity were nonsignificant (*b* = .01, *p* = .49; b = −.01, *p* = .92 respectively).

### Number of fixations E:M

**Figure 3.**
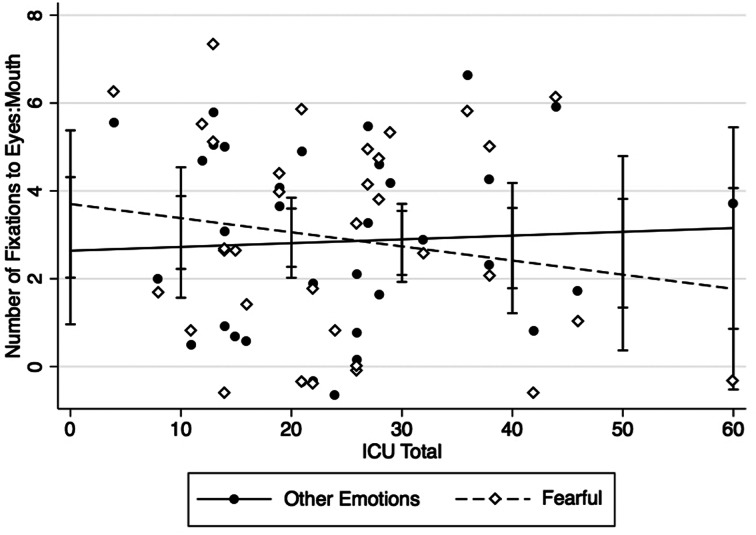
Callous-unemotional (CU) traits in adolescents with autism spectrum disorder (ASD are associated with decreased number of fixations to the eyes versus mouth when viewing fearful faces

There was no main effect of emotion (*b* = .06, *p* = .77) or CU traits (b = .01, *p* = .93). The interaction term (CU-by-emotion) was significant (β = −.04, *p* = .02; Figure 3), indicating less attention towards the eye as compared to mouth area in those with CU traits when viewing fearful faces. There was no effect of IQ or ASD severity (*b* = −.01, *p* = .64; *b* = −.02, *p* = .92, respectively). To clarify what was driving the association with E:M ratio, associations between CU traits and the number of fixations to the eyes and the number of fixations to the mouth (both divided by the total number of fixations during stimuli presentation) were tested separately. There was a trend association between the CU-by-emotion interaction term and number of fixations to the eyes (*b* = −.01, *p* = .07), suggesting fewer looks to the eyes when viewing fearful faces in those with CU traits. The association with number of fixations to the mouth was nonsignificant (*b* = .01, *p* = .63).

### Associations between visual attention and task performance

No significant correlations were found between total looking time E:M and accuracy (*r* = .10, *p* = .56) or RT (*r* = −.05, *p* = .76), or between number of fixations E:M and accuracy (*r* = .20, *p* = .25) or RT (*r* = .04, *p* = .81).

## Discussion

This study describes the prevalence and associated cognitive profile of CU traits in youth with ASD. We have reported on this topic previously (Carter Leno et al., 2015); the current study represents a replication and extension of prior work in an independent sample. We use a more psychometrically robust measure to assess CU traits and find 22% of the current sample score above the threshold expected to identify the top 6% of scorers. As expected, CU traits were associated with higher levels of conduct and peer problems, and lower levels of prosocial behavior. We used a novel experimental task to test the association between CU traits and emotion recognition. We find CU traits are associated with longer RTs and fewer fixations to the eyes as compared to the mouth when viewing fearful faces. No associations are found between CU traits and accuracy to identify fearful faces, or total time spent looking at the eyes as compared to the mouth.

Although the current estimate of prevalence is lower than previously reported in ASD samples (e.g., 51% in Carter Leno et al., 2015; 36% in Rogers et al., 2006), previous work has not reported 95% CIs so comparison between estimates should be read with caution. Nonetheless, the current results (22%) still indicate a four-fold increase from prevalence rates of CU reported in non-ASD youth (Collishaw et al., 2004; Fontaine et al., 2011). This increase in prevalence of CU traits is of note given the overall ASD population from which the sample was drawn; a community-based sample not purposely enriched for behavioral problems or other difficulties. Indeed, the percentage of those scoring above threshold for “definite” conduct problems (30%; as identified by the SDQ) is in line with existing prevalence estimates of disruptive behavior problems in youth with ASD (see Kaat & Lecavalier, 2013 for a review), including those who use population-representative samples (Simonoff et al., 2008).

Differences in the estimated prevalence of CU traits in ASD samples are likely in part due to differences in the instrument used. Previous literature has often used the APSD (Frick & Hare, 2001) to asses CU traits, which are measured by one subscale with six items. The limited number of subscale items likely contributes to the moderate internal consistency reported in typically developing (α = .56 in Dadds et al., 2005) and ASD samples (α = .59 in Carter Leno et al., 2015). Issues of assessment validity may be especially pertinent, as some behaviors associated with CU traits can be superficially similar to features of ASD (such as lack of sensitivity to the feelings of others), therefore instruments designed to measure CU traits in typical populations may not be sensitive enough to discriminate between overlapping aspects of ASD and CU traits. These issues of assessment validity and construct overlap of CU traits and ASD mean that the result of increased prevalence of CU traits in ASD as compared to non-ASD populations should be interpreted with caution. Although the current study used the ICU, which has more items and good internal consistency in the current sample, this does not rule of the possibility that the reported increase in prevalence still partly reflects measurement difficulties. One way to avoid these issues is to develop ASD-specific questionnaires, which may better discriminate between CU and ASD-type behaviors. However, this then prohibits any comparisons with estimates from non-ASD samples.

In contrast to previous work (Carter Leno et al., 2015), we found a significant increase in the proportion of individuals with high conduct problems in the high CU trait group. Forty-four per cent of the high CU group scored above threshold for conduct problems, as compared to 16% in the low CU group. However, the strength of association is still lower than that reported in general population samples, where those with CU traits had a 0.95 probability of having concomitant conduct problems (Fontaine et al., 2011). This apparent dissociation of CU traits from conduct problems in ASD populations may indicate a different cognitive underpinning, or, as previously suggested (Carter Leno et al., 2015), it could also be possible that the socio-behavioral differences associated with ASD may prevent individuals from engaging in the more socialized features of conduct-disordered behavior. In continuous analyses, we replicate previously reported associations between CU traits and conduct problems, peer problems and prosocial behavior (Carter Leno et al., 2015), which remain after adjusting for ASD severity, suggesting these traits have a selective impact on the behavioral presentation of ASD beyond difficulties driven by core autism severity. Results reinforce the notion that it is important to consider the contribution of CU traits to the heterogeneous behavioral presentation of ASD, as certain behavioral problems could be being misinterpreted as being solely due to ASD. We found no association between CU traits and sex, raising the question of whether certain factors thought to increase risk for psychopathology in the general population function differently in ASD (Simonoff et al., 2008).

In line with that reported from non-ASD samples (Moffitt & Silva, 1988), we found a stronger association with verbal as compared to performance IQ. We also replicate the association between CU traits and fear recognition (Carter Leno et al., 2015), as is found in non-ASD populations (Marsh & Blair, 2008; Muñoz, 2009; White et al., 2016). However, as our analytic approach collapsed all other emotions into one category to maximize statistical power in a moderately sized sample, we did not test whether recognition of any other emotions showed an association with CU traits. It should be noted that previously work with larger ASD samples which tested each emotion individually only finds impairment in fear recognition (Carter Leno et al., 2015), although others also report impairments for sadness (Rogers et al., 2006). Ceiling effects may have contributed to the lack of association between CU traits and accuracy of emotion recognition. We chose to use a matching emotion recognition task to maximize participation; however, this may have inadvertently led to a less sensitive test of emotion recognition. RT may represent a more sensitive measure of performance on this task.

Crucially, extending our previous work, we found CU traits were associated with fewer fixations to the eye as compared to the mouth area during viewing of fearful faces, and this result appeared to be driven by fewer looks to the eyes, as in non-ASD populations (Dadds et al., 2006, 2008). One could conclude that problems in attentional cuing are likely to underlie the difficulties in fear recognition associated with CU traits in individuals with ASD, as has been proposed in individuals without ASD. However, we did not find any association between visual attention and task performance. It could be that by using more ecologically valid (and thereby less controlled) stimuli, performance on the task was not only reliant upon information gleaned from the eyes and mouth, but also from the rest of the face (which was not measured by our AOIs).

The lack of association with gaze duration is unexpected; previous work in non-ASD populations reports a decrease in both the total duration and number of fixations to the eyes in those with CU traits (Dadds et al., 2008). One explanation is that individuals with ASD are known to have difficulties disengaging visual attention once it has been captured (Landry & Bryson, 2004). Therefore, more looking time to the eyes or mouth may not reflect the same thing in ASD versus non-ASD populations. It may be that in the current sample, once attention was captured, participants remained looking at that area due to difficulties switching attention, rather than any intrinsic preference. Thus, number of fixations to a given area may be a stronger indicator of preference, as this would require participants to move their gaze away from the area and then return.

The next step in this line of research is to assess the directionality of associations between visual attention, recognition of fear and CU traits in ASD populations. In non-ASD samples, reduced looking to the face at 5 weeks predicts CU traits later in childhood (Bedford et al., 2015), suggesting differences in orienting to social stimuli early in infancy may represent a key developmental mechanism. Similarly, there is some evidence to suggest early differences in attention to the eyes predict later ASD outcome (Jones & Klin, 2013; although see Kwon et al., 2019). Whether early decreases in attention to social stimuli represent a shared pathway in the development of both CU traits and ASD (Dadds & Frick, 2019), and how a “double hit” of the two may manifest in early infancy is an important topic for future research as it may elucidate why the rates of CU traits are elevated in ASD populations. The mechanism of co-occurrence may be that of a “direct pathway”, in that emerging ASD leads to decreased time looking to the eyes, which in turn has a knock-on effect on emotion recognition ability and increases the likelihood for CU traits. However, it is also possible that ASD and CU are associated with independent genetic influences (Jones et al., 2009), but converge on a similar cognitive pathway, leading to certain superficially similar impairments, in addition to other disorder-specific pathways. It should also be acknowledged that although both disorders are associated with less looking towards the eyes, the information gleaned might be being processed differently. Combining eye-tracking and neuroimaging techniques would shed light on similarities in gaze processing between the two groups.

### Strengths and limitations

The current study has several strengths. First, our prevalence estimates and tests of behavioral associations utilized a large well-characterized community-based sample of individuals with ASD, enriched for females, with a wide range of ability. We acknowledge that although we present prevalence estimates, to give a true estimate of the prevalence of CU traits in adolescents with ASD a fully population representative ASD sample is required. Although the current sample is community-based, and therefore likely less biased towards complex cases than if we had recruited adolescents currently attending a clinical service, we relied on clinical records to identify cases (i.e., individuals with a diagnosis of ASD) to invite into the study when participants were aged 4–8 years old. This means that any individuals who may have been true ASD cases, but for various reasons were undetected or had not received a clinical diagnosis by the services from whom we recruited upon time of study recruitment, were not included in the sample.

Although we had a large sample for the estimation of prevalence and behavioral correlates of CU traits, only a subsample completed the emotion recognition task. Being aware of our moderately sized sample, we restricted our analyses to testing performance on fearful versus all other emotions to limit multiple comparisons. We also used a novel and more ecologically valid task, combined with eye-tracking, to test the association between CU traits and emotion recognition, thus expanding previous work which has primarily used static stimuli and only investigated behavioral performance. However, as this task is novel, it is not yet well-used, therefore findings require further replication.

## Conclusions

Current findings provide further support for the notion that CU traits are elevated in individuals with ASD and are associated with a more severe behavioral presentation. Clinicians may benefit from considering the role of CU traits when assessing individuals with ASD who present with conduct problems, although issues of construct overlap should be held in mind. Results also suggest that CU traits may share a similar etiology in people with and without ASD. Future work should assess the directionality of associations between visual attention, affective processing and CU traits over time in individuals with and without ASD.

## Supplementary material

For supplementary material accompanying this paper visit https://doi.org/10.1017/S0954579420000449. click here to view supplementary material
