# Supplementary material for: Callous-unemotional traits in youth with autism spectrum disorder (ASD): replication of prevalence estimates and associations with gaze patterns when viewing fearful faces
Source: Dev Psychopathol. 2021 Oct;33(4):1220–8. doi: 10.1017/S0954579420000449 (PMC8564715; doi:10.1017/S0954579420000449)
Supplement: Supplementary file 1 [file S0954579420000449sup001.docx]

**Supplementary Materials**

**Sample**

The original target population for the study was all children born in a four-year period (01/09/2000 and 31/08/2004), living in two London boroughs (inner/outer London), who had a clinical diagnosis of ASD (see Supplementary Figure 1). Clinical diagnosis was established following a multidisciplinary neurodevelopmental and social communication assessment led by a single community paediatrician for each borough.

As recommended by the project’s lead statistician, the ADI-R was administered at Wave 2 to intensive sample participants if a) twice the ADOS-2 score plus the SCQ score lay between 12 and 46 inclusive or, b) if twice the ADOS-2 algorithm score minus the SCQ score was less than or equal to -5, or was greater than or equal to 17. This was to capture participants who were not being rated highly by the combined SCQ and ADOS-2 scores, or those in which there was a large discrepancy between the SCQ and the ADOS-2 scores. The ADI-R was required in 66/83 cases. Both the autism cut-offs recommended by Rutter et al. (2003) and the ASD cut-offs recommended by Risi et al. (2006) were applied to the ADI-R data. All participants were above the diagnostic cut-offs for autism or autism spectrum disorder on either or both the ADOS-2 and the ADI-R.

**Participated in Wave 1**

N = 277 (62.0% of target population)

82.0% male

Selected for Wave 1 Intensive assessment

n=131

Did not participate in Intensive assessment

Assessment completed

**Intensive Sample**

N=101

56% male

**Extensive Sample**

N=176

97% male

**Eligible for Wave 2**

N=277 (all Wave 1 ppts)

**Extensive sample participation**

N= 128 (73% of Wave 1 ppts)

**Intensive sample participation**

N= 83 (82% of Wave 1 ppts)

**Completed eye-tracking assessment**

N= 46 (55% of Wave 2 intensive sample)

**Target population**

Children with an ASD diagnosis, born 01/09/2000- 01/09/2004, living in Bromley or Lewisham

N=447

**Supplementary Figure 1. Summary of QUEST Sample Recruitment and Selection**

**Additional Information About Emotion Recognition Paradigm**

Trials consisted of an initial fixation cross on a scrambled background (2.5 seconds), followed by a dynamic video of an actor portraying a specific emotion (1.5 seconds of motion, followed by 1 second freeze-frame static image of the expression), a centrally presented fixation cross on the same scrambled background (1 second), and then a response screen displaying four static pictures of different actors portraying different emotions (8 seconds). To create the dynamic videos, four female actors were filmed performing different emotional expressions, keeping distance from camera matched across actors. The scrambled background for each trial was a luminance-matched shuffled pixel version of the freeze-face of the emotional expression presented after the dynamic video. These backgrounds were created to eradicate afterimages. The only change from the current paradigm to the original developed by Bedford and colleagues was changing the initial fixation cross from being centrally presented to instead being presented on the top of the screen for 50% of trials and at the bottom of the screen for 50% of trials.

**References**

Rutter, M., Le Couteur, A., & Lord, C. (2003). *Autism Diagnostic Interview-Revised*. Los Angeles, CA: Western Psychological Services.

Risi, S., Lord, C., Gotham, K., Corsello, C., Chrysler, C., Szatmari, P., . . . Pickles, A. (2006). Combining information from multiple sources in the diagnosis of autism spectrum disorders. *Journal of the American Academy of Child & Adolescent Psychiatry, 45*(9), 1094-1103.
